# p38 MAPK Regulates Cavitation and Tight Junction Function in the Mouse Blastocyst

**DOI:** 10.1371/journal.pone.0059528

**Published:** 2013-04-04

**Authors:** Christine E. Bell, Andrew J. Watson

**Affiliations:** 1 Department of Obstetrics and Gynaecology, The University of Western Ontario, London, Ontario, Canada; 2 Department of Physiology and Pharmacology, The University of Western Ontario, London, Ontario, Canada; 3 Children's Health Research Institute, London, Ontario, Canada; 4 Lawson Health Research Institute, London, Ontario, Canada; Laboratoire de Biologie du Développement de Villefranche-sur-Mer, France

## Abstract

Blastocyst formation is essential for implantation and maintenance of pregnancy and is dependent on the expression and coordinated function of a series of proteins involved in establishing and maintaining the trans-trophectoderm ion gradient that enables blastocyst expansion. These consist of Na/K-ATPase, adherens junctions, tight junctions (TJ) and aquaporins (AQP). While their role in supporting blastocyst formation is established, the intracellular signaling pathways that coordinate their function is unclear. The p38 MAPK pathway plays a role in regulating these proteins in other cell types and is required for embryo development at the 8–16 cell stage, but its role has not been investigated in the blastocyst.

**Hypothesis:**

p38 MAPK regulates blastocyst formation by regulating blastocyst formation gene expression and function.

**Methods:**

Embryos were cultured from the early blastocyst stage for 12 h or 24 h in the presence of a potent and specific p38 MAPK inhibitor, SB 220025. Blastocyst expansion, hatching, gene family expression and localization, TJ function and apoptosis levels were analyzed.

**Results:**

Inhibition of the p38 MAPK pathway reduced blastocyst expansion and hatching, increased tight junction permeability, affected TJP1 localization, reduced *Aqp3* expression, and induced a significant increase in apoptosis.

**Conclusion:**

The p38 MAPK pathway coordinates the overall events that regulate blastocyst formation.

## Introduction

Mammalian preimplantation development extends from fertilization to implantation and culminates in the formation of a blastocyst, a fluid filled structure that emerges from the zona pellucida and implants into the uterine wall to maintain pregnancy [1], [2]. The early blastocyst consists of two cell types, the inner cell mass (ICM) and the trophectoderm (TE) [1], [2]. The ICM is an undifferentiated mass of cells that will become the embryo proper. The TE is a polarized epithelial cell layer which surrounds the ICM. The TE mediates implantation to the uterine wall, contributes to the embryonic portion of the placenta and is characterized by the expression of transcription factors (TF), such as caudal homeobox two (*Cdx2*) [3]. Functional adherens junctions (AJ) and tight junctions (TJ) form a seal between the cells of the TE, which is essential for fluid accumulation and formation of the blastocyst cavity [4]–[12].

The Na/K ATPase is also a critical mediator of blastocyst formation as it establishes a trans-trophectoderm ionic gradient that directs fluid movement across the TE epithelium [13]–[18]. The Na/K ATPase is comprised of two main subunits; the alpha (ATP1A) and the beta (ATP1B). Loss of *Atp1a* during preimplantation development does not impede the initial formation of a blastocyst, but shortly after results in an embryonic lethality during peri-implantation development [19]. In contrast, loss of *Atp1b1* results in a developmental arrest at the morula stage, and blastocyst expansion does not occur [18]. Additionally, the Na/K ATPase acts as a ouabain mediated signaling molecule that regulates TJ formation and function in the TE [8], [12]. Fluid movement across the TE is facilitated by the presence of aquaporins (AQP) 3 and AQP 9 in the TE membrane [19]. AQP 9 is localized to the apical surface of the TE and AQP 3 is localized to the baso-lateral surface of the TE, and together, they facilitate fluid movement from the ‘outside’ to the ‘inside’ of the blastocyst cavity, along the ionic gradient established by the baso-laterally localized Na/K ATPase [19].

Together, the AJs, TJs, Na/K ATPase and AQPs, are critical gene products that coordinate blastocyst expansion and blastocyst cavity formation (reviewed in [2]). Although the cell biology of blastocyst formation is well documented, the molecular signaling events, particularly the intracellular signaling events, governing blastocyst formation are unclear (reviewed in [2], [20]). The p38 MAPK pathway is an intracellular signaling pathway that translates extracellular stimuli into cellular responses [21]. This intracellular signaling pathway directs a wide variety of physiological processes through Serine/Threonine phosphorylation [21]. p38 MAPK is ubiquitously expressed in all eukaryotic cells and regulates gene expression, mitosis, migration and apoptosis [21]–[25]. Each component of the p38 MAPK pathway is present throughout preimplantation development and the p38 MAPK pathway plays both developmental and adaptive roles during preimplantation development [22], [25]–[27]. Our previous studies have demonstrated that p38 MAPK is both an important mediator of early preimplantation development and is also a component of the adaptive mechanism the embryo can employ to adjust to environmental influences during development [22], [25]–[27]. p38 MAPK is a gene family consisting of four different isoforms, α, β, γ and δ [21]. Our previous studies focusing on the role of p38 MAPK during preimplantation development demonstrated that treatment of 2-, 4- and uncompacted 8-cell embryos with the cytokine suppressive anti-inflammatory drugs (CSAIDs^TM^; SB203580 and SB220025 active forms; SB202474 inactive form control) all result in a reversible developmental blockade that suspends development at the 8–16 cell stage [22], [26]. SB203580 and SB220025 are potent and specific pharmacological agents that are routinely and reliably used to investigate p38 MAPK function in cell systems (reviewed in [21], [28]. We also demonstrated that the p38 MAPK inhibited blockade is accompanied by a downstream loss of MAPKAPK2/3 phosphorylation, then loss of HSP25/27 phosphorylation and finally a disassembly of filamentous actin [22], [26]. 8–16 cell stage drug free embryos resume their developmental program and progress normally to the blastocyst stage following treatment with an accompanying delay consistent with the original inhibitor treatment time [22], [26].

In addition, the p38 MAPK pathway directs preimplantation embryonic responses to environmental stress [25], [27]. Fong *et al*., 2007 reported that MAPKAPK2, a downstream target of p38 MAPK, displayed enhanced phosphorylation in embryos exposed to hyperosmotic culture medium. This elevation in MAPKAPK2 phosphorylation was extinguished when the embryos were cultured under hyperosmotic conditions in the presence of SB 220025, demonstrating that p38 MAPK was activated in response to hyperosmotic stress [27]. Bell *et al*., 2009 demonstrated that the p38 MAPK pathway up-regulated *Aqp3* and *Aqp9* mRNA expression in response to hyperosmotic stress in 8-cell embryos but did not regulate blastocyst apoptosis levels resulting from hyperosmotic stress [25]. Taken together, these results demonstrate that p38 MAPK signaling regulates both the developmental program of preimplantation development and embryonic adaptations to environmental stress.

The present study was conducted to investigate the role of p38 MAPK in regulating cavitation and blastocyst formation. Embryos were cultured from the early blastocyst stage for 12 h or 24 h in the presence of a p38 MAPK inhibitor, SB 220025. Blastocyst expansion, zona hatching, TE formation and function, and apoptosis levels were assessed. We have demonstrated that the p38 MAPK pathway is required for blastocyst expansion and hatching and for regulating TJ function. Furthermore, suppression of p38 MAPK signaling resulted in a significant increase in apoptosis levels. These results demonstrate that p38 MAPK also exerts its influences on blastocyst formation and suggests that it is an important mediator of blastocyst formation gene family expression, function and thus coordination of the overall events that regulate blastocyst formation.

## Results

### Reduced expansion and hatching in p38 MAPK inhibited embryos

**Figure 1:**
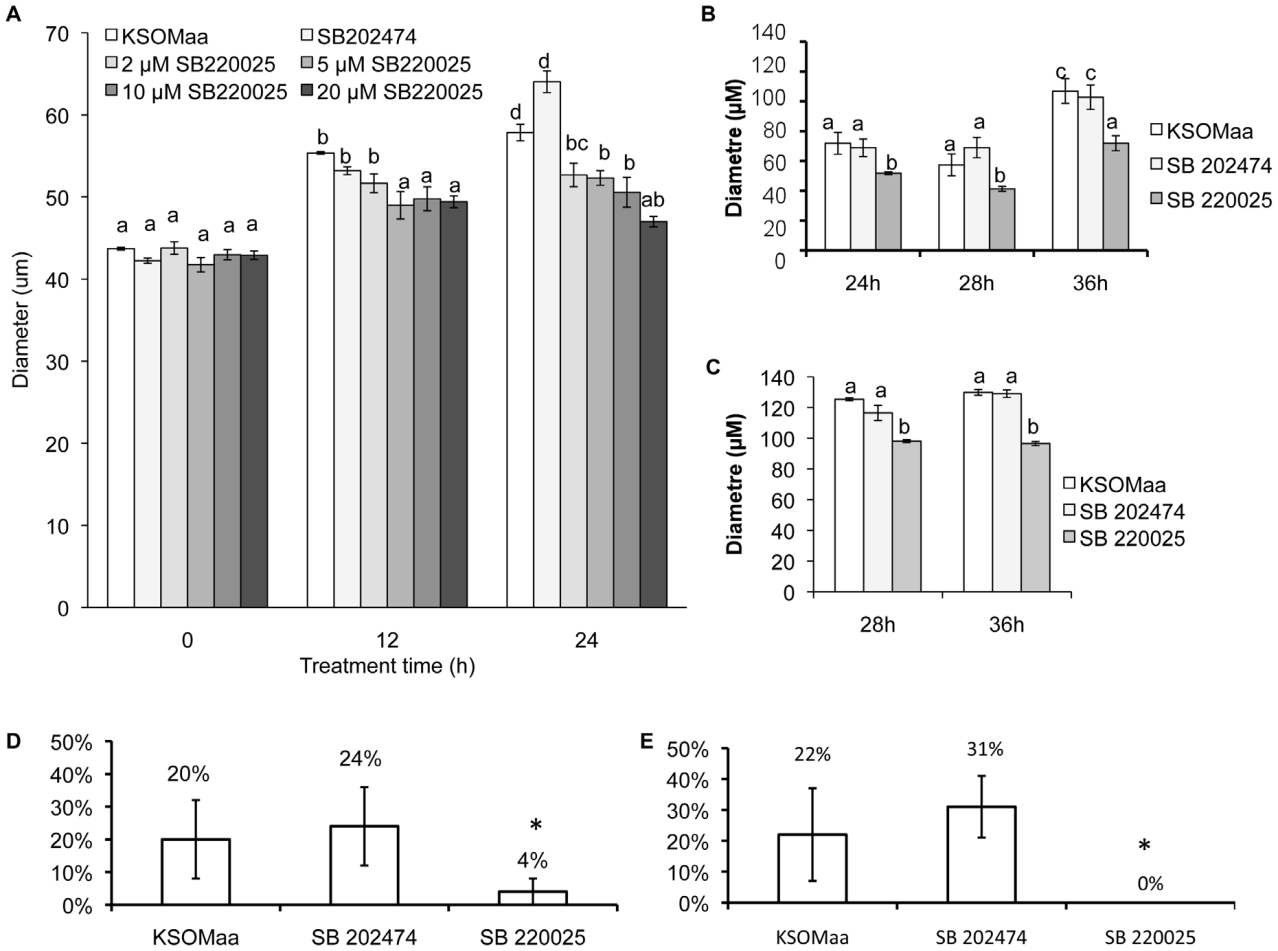
p38 MAPK Regulates Blastocyst Expansion and Hatching. p38 MAPK inhibition decreased embryo expansion and hatching. (A) After 12 h and 24 h embryos cultured in KSOMaa + SB 220025 were significantly less expanded than controls. Three replicates were performed and 30–35 embryos were measured in each group. SEM p≤0.05; letters (a–c) represent groups that are significantly different from one another. (B–C) Embryo recovery after 12 h (B) and 24 h (C) treatment of SB 220025. Embryos were washed in fresh KSOMaa after 12 h or 24 h and allowed to recover until 24 h, 28 h, and 36 h post collection. Embryos treated with SB 220025 for 12 h remained significantly less expanded compared to controls, however, they resumed cavitation and increased their expansion following treatment. Embryos treated with SB 220025 for 24 h remained significantly less expanded compared to controls, and were not able to re-expand. Three replicates were performed and 30–35 embryos were measured in each group. SEM p≤0.05; letters (a–c) represent groups that are significantly different from one another. (C) Inhibition of the p38 MAPK pathway blocked embryo hatching. Embryos cultured in SB 220025 for 12 h or 24 h displayed significantly reduced zona hatching compared to controls. Embryos were cultured in KSOMaa, SB 202474 or SB 220025 for 12 h and then washed in fresh KSOMaa and cultured until 24 h post collection and assessed for hatching. Embryos that had begun to hatch were counted as positive (SEM; p≤0.05; *n* = 4).

We first defined the concentration and time course effects of treating early blastocysts with the p38 MAPK inhibitor SB 220025 (Figure 1A). Embryos cultured in all tested concentrations of SB 220025 did not display a significant difference from control embryos following 4 h and 8 h of treatment (data not shown). After 12 h of treatment, embryos cultured in 5 µM SB 220025 (diameter, 48.99 microns ±0.88 microns), 10 µM SB 220025 (49.77 microns ±0.67 microns) or 20 µM SB 220025 (49.41 microns ±0.30 microns) displayed a significant reduction in blastocyst diameter compared to control groups: KSOMaa +0.2% DMSO (54.87 microns ±0.62 microns), KSOMaa +20 µM SB 202474 (inactive analogue of SB 220025) (53.20 microns ±2.37 microns). After 24 h of culture, embryos cultured in 2 µM SB 220025 (52.68 microns ±2.04 microns), 5 µM SB 220025 (52.31microns ±1.18 microns), 10 µM SB 220025 (50.57 microns ±2.12 microns) or 20 µM SB 220025 (47.01 microns ±0.84 microns) all displayed a significant reduction in blastocyst diameter compared to control groups: KSOMaa +0.2% DMSO (61.14 microns ±1.34 microns), KSOMaa +20 µM SB 202474 (64.04 microns ±0.56 microns) (Figure 1; Figure S1). From these data, we selected 5 µM SB 220025 as the lowest effective concentration for investigating the effects of p38 MAPK inhibition on blastocyst formation and used this concentration in all subsequent experiments. To validate the specificity and reproducibility of these outcomes, we repeated this experiment using a second p38 MAPK inhibitor, SB 203580. Our outcomes employing 5 µM SB 203580 for 24 h were identical to the SB 220025 outcomes in that we observed a significant reduction in blastocyst diameter in the presence of SB 203580 (Figure S2). To ensure the effects we were observing were not being mediated by inadvertently affecting the ERK MAPK pathway, we probed embryos treated for 24 h with 5 µM SB 220025 for total-ERK and phospho-ERK levels by immunofluorescence. Treatment with SB 220025 had no observable influence on total or phospho-ERK fluorescence levels (Figure S3).

After 12 h and 24 h of treatment, embryos were washed extensively in fresh, drug free medium and then placed into fresh culture drops for a treatment recovery period. Embryos treated for 12 h and then placed into recovery medium remained significantly less expanded than controls; however, they did resume expansion and continued to expand for the full recovery period (Figure 1B). Embryos treated for 24 h with SB 220025 did not resume blastocyst expansion following this treatment (Figure 1C).

As we established that p38 MAPK signaling regulates blastocyst expansion, we next investigated the role of p38 MAPK signaling in blastocyst hatching *in vitro*. Zona hatching was assessed at 24 h and 48 h post collection. Blastocysts treated for 12 h (Figure 1D) with SB 220025 displayed reduced zona hatching (4%±4%) compared to KSOMaa (20%±15%) and SB 202474 (24% ±10%) controls at 24 h post collection. Blastocysts treated for 24 h (Figure 1E) with SB 220025 (0%) displayed a significant (p≤0.05) reduction in zona hatching compared to controls; KSOMaa (22%±12%) or SB 202474 (31%±10%). Extending the experimental period to 48 h post collection (i.e. 12 h or 24 h treatment followed by 36 h or 24 h recovery respectively), had no benefit as embryos treated with SB 220025 remained enclosed by the zona pellucida (data not shown).

### Blastocyst expansion failure is accompanied by increased TJ permeability and disrupted localization of Tjp1

**Figure 2:**
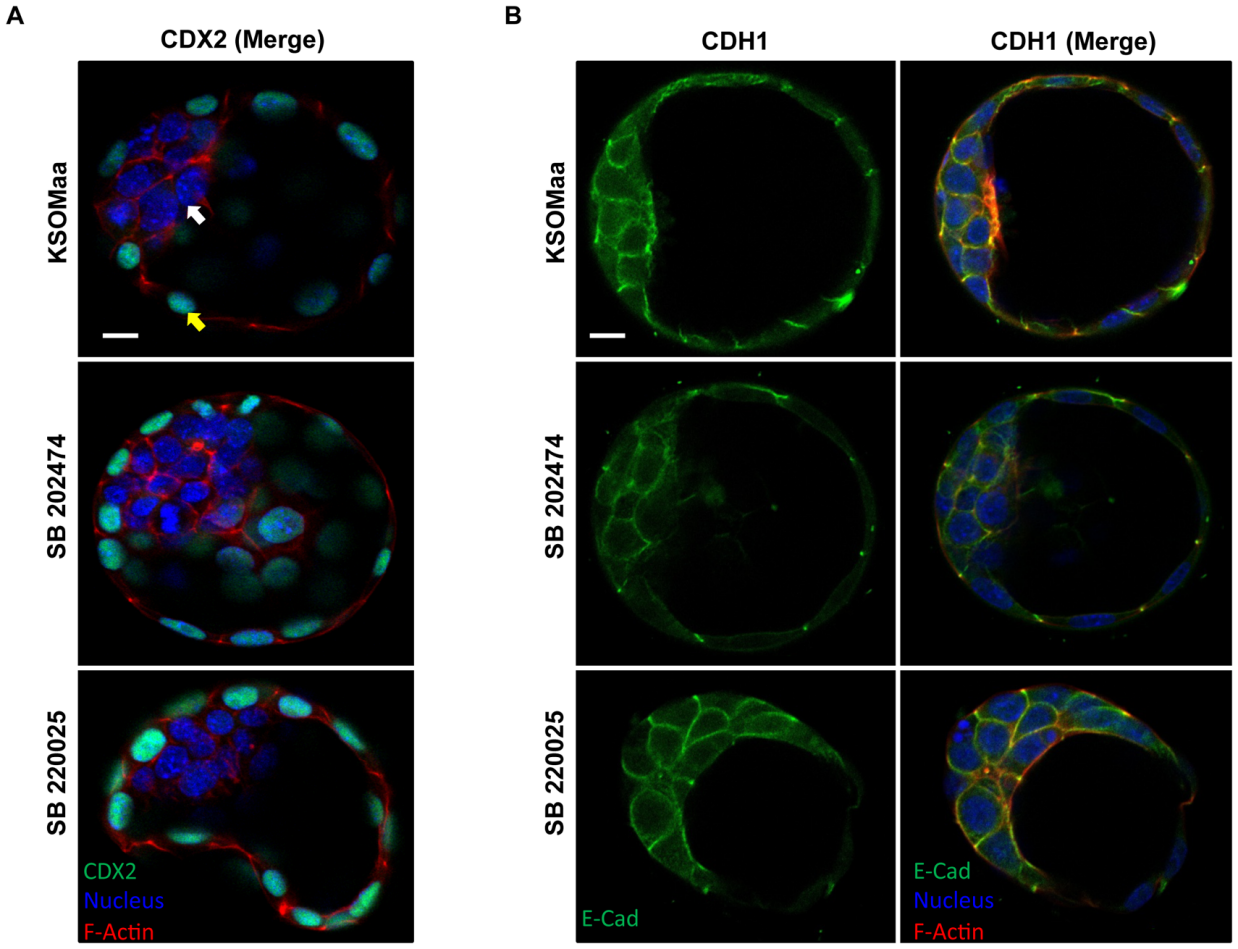
TE formation and apico-basal cell polarity in TE appear normal in p38 MAPK inhibited embryos. CDX2 protein localization (A) following 24 h of treatment with KSOMaa, SB 202474, or SB 220025. White arrows indicate ICM; Yellow arrows indicate TE. Blue  =  nucleus; Red  =  F-actin; Green  =  CDX2; n = 10–15 embryos in each group; scale bar  = 10 microns. CDH1 protein localization (B) following 24 h of treatment with KSOMaa, SB 202474, or SB 220025. CDH1 protein localization pattern did not vary after 24 h. Blue  =  nucleus; Red  =  F-actin; Green  =  CDH1; *n* = 10–15 embryos in each group; scale bar  = 10 microns.

Since p38 MAPK inhibition resulted in reduced blastocyst expansion, we wanted to investigate whether this was linked to impaired TE differentiation or improper TE polarization (Figure 2). To assess TE differentiation, we investigated the expression of CDX2, a TF that is required for TE lineage specification [3]. CDX2 localization was maintained and confined to the outer cells (yellow arrows) of both control and SB 220025 treated blastocysts (Figure 2A). To investigate TE polarization, we characterized the localization of CDH1, which is normally localized to the basolateral membranes of the TE (reviewed in [2], [20]). CDH1 maintained a normal, basolateral cell margin distribution pattern in the TE cells in each treatment group (Figure 2B). These results support the conclusion that TE differentiation and polarization were not affected by treatment with SB 220025.

### p38 MAPK Inhibition Compromises TJ Permeability

**Figure 3:**
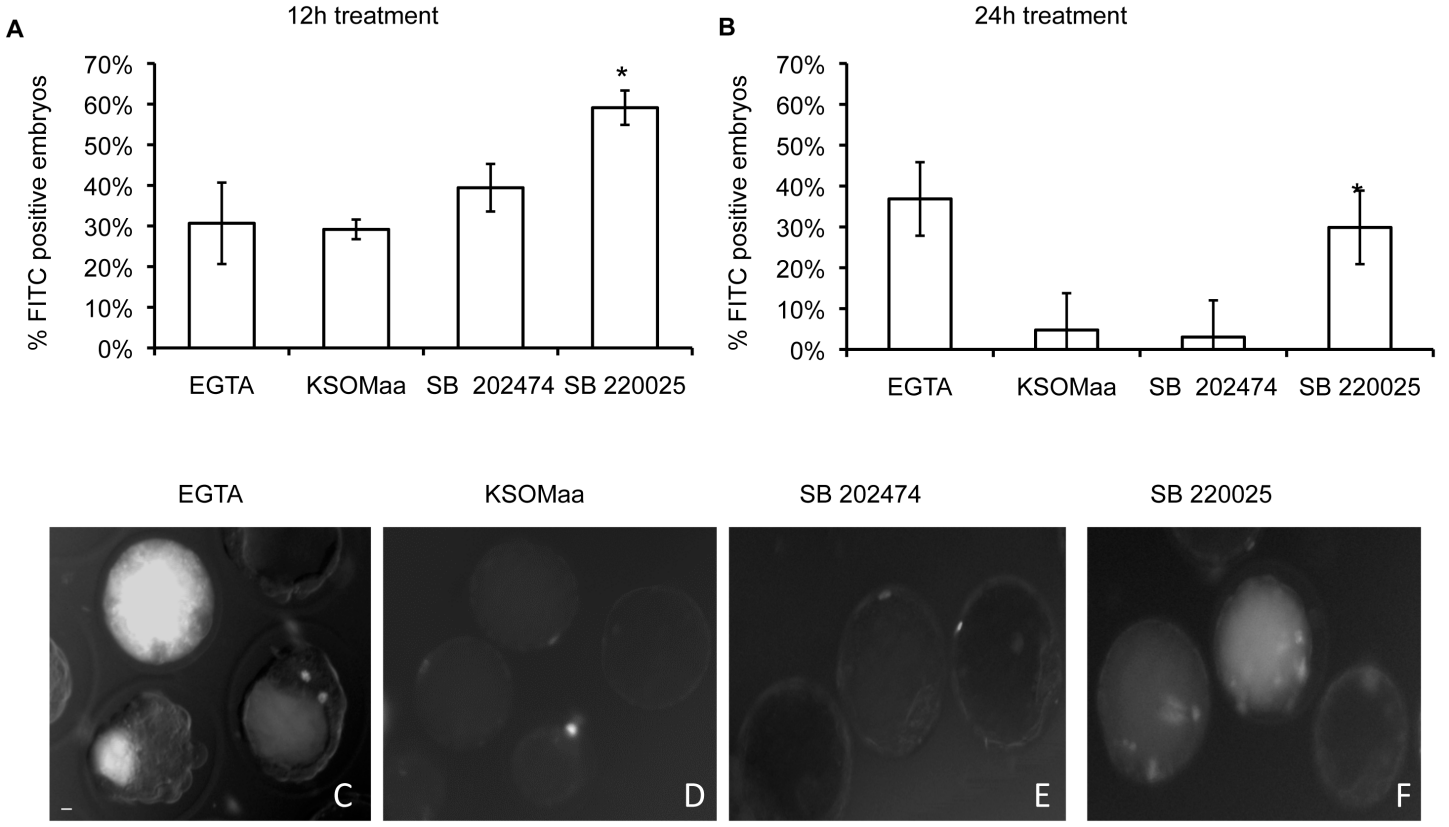
p38 MAPK inhibition compromises TJ integrity. To test TJ integrity, embryos were treated in KSOMaa, SB 202474, or SB 220025 then exposed to 40 kDa FITC-dextran. After 12 h of treatment (A), a significant difference was observed in the number of embryos displaying 40 kDa FITC-dextran fluorescence within the blastocyst cavity between SB 220025 and control groups (p≤0.05) n = 3. After 24 h of treatment (B), a significant difference was observed in the number of embryos displaying 40 kDa FITC-dextran fluorescence within the blastocyst cavity between SB 220025 and control groups (p≤0.05) n = 3. Representative images of embryos following 12 h or 24 h treatment with (C) 2 mM EGTA (positive control), (D) KSOMaa, (E) SB 202474 or (F) SB 220025 using 40 kDa FITC-dextran. *n* = 3; ± SEM; scale bars  = 10 microns.

**Figure 4:**
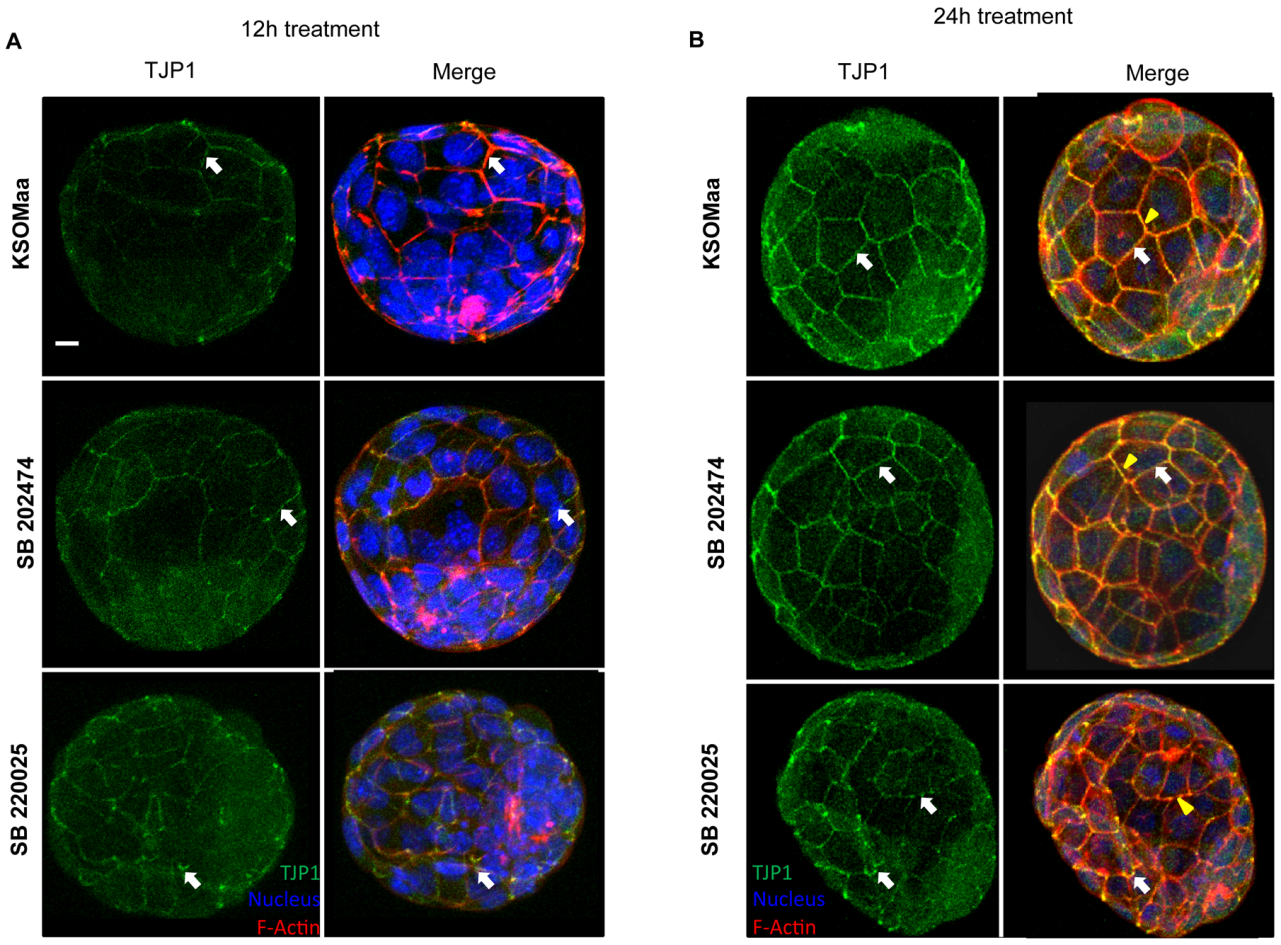
Inhibition of p38 MAPK affects TJP1 localization after 12 h and 24h. TJP1 protein localization following treatment with KSOMaa, SB 202474, or SB 220025. Representative images of TJP1 after 12 h (A) and 24 h (B) exposure. SB 220025 treatment affected TJP1 localization after 12 h (A) and 24 h (B). In KSOMaa and SB 202474 images, white arrows indicate normal localization of TJP1 along adjacent cell borders. In p38 MAPK inhibited images, white arrows indicate punctate distribution of TJP1 compared to control embryos. In control embryos, yellow staining indicates co-localization of TJP1 and F-actin (yellow arrowhead). In treated embryos, there is reduced yellow staining, and increased red staining indicating a loss of co-localization of TJP1 and F-actin (yellow arrowhead). Blue  =  nucleus; Red  =  F-actin; Green  =  TJP1; *n* = 10–15 embryos in each group; scale bar  = 10 microns.

Since blastocyst cavitation was reduced in p38 MAPK inhibited embryos, we next sought to investigate whether TE permeability was compromised in SB 220025 treated embryos. We investigated effects of SB 220025 treatment on TJ permeability using a standard FITC-dextran assay (Figure 3) [8], [12]. Blastocyst expansion is dependent upon the formation of a TJ permeability seal in the TE (reviewed in [2], [20]). Changes in the permeability of this TJ seal can be precisely assessed by applying a sensitive FITC-Dextran assay [8], [34]. Following 12 h (Figure 3A) and 24 h (Figure 3B) SB 220025 treatment, we observed a significant increase in FITC-dextran accumulation into the blastocyst cavity compared to controls. This demonstrated that p38 MAPK activity regulates TE TJ permeability. We then assessed effects of treatment on TJP1 localization. Blastocysts treated for 12 h (Figure 4A) with SB 220025 displayed abnormal TJP1 cortical staining compared with control embryos. TJP1 is normally distributed along the apico-baso membrane in a smooth, continuous pattern as shown in control embryos cultured in KSOMaa or SB202474 (Figure 4A). TJP1 also co-localizes with the cytoskeleton, which can be observed as a yellow signal in the merged images of both KSOMaa and SB 202474 treated embryos (Figure 4A; KSOMaa merged image; SB 202474 merged image). In p38 MAPK inhibited embryos, TJP1 appears in tight foci at cell-cell contacts with punctate staining and there is a loss of yellow co-localization signal revealing a predominantly red signal, indicative of F-actin signal alone and a loss of TJP1 co-localization (Figure 4A; SB220025 merged image). The same punctate distribution along the apico-baso membrane and loss of yellow co-localization signal was observed in embryos treated after 24 h (Figure 4B) of p38 MAPK inhibition. After 24 h, TJP1 localization was considerably more punctate along adjacent cell borders in SB 220025 treated embryos compared to 12 h of treatment and compared to control embryos. This punctate distribution was observed in 70% of embryos treated for 12 h and 85% of embryos treated for 24 h with SB 220025 as scored by two independent observers.

**Figure 5:**
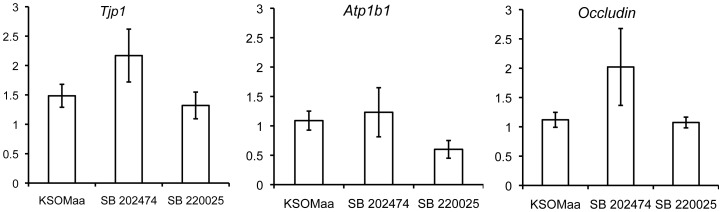
p38 MAPK inhibition does not affect mRNA expression of *Tjp1*, *Atp1b1*, or *Occludin*. q-PCR was utilized to determine mRNA expression levels of *Tjp1, Atp1b1,* and *Occludin.* There was no significant difference in mRNA expression levels between treatment groups. (*n* = 4, mean ± SEM).

**Figure 6:**
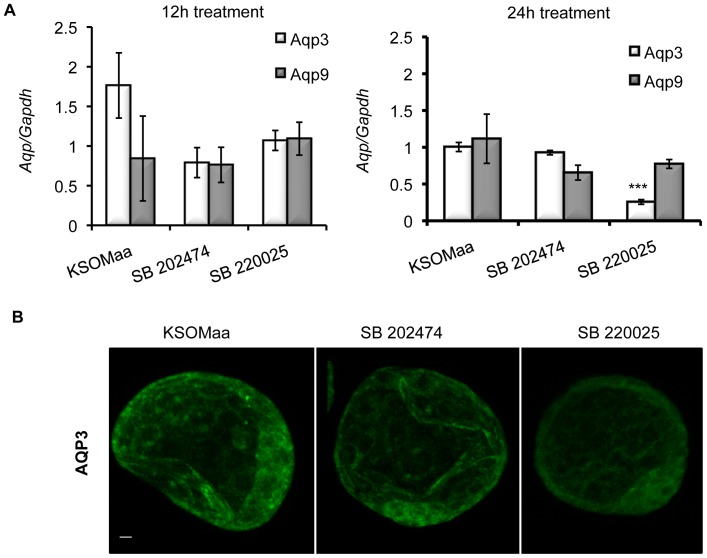
Inhibition of p38 MAPK affects *Aqp3* mRNA expression and protein localization. (A) Relative *Aqp 3* and *9* mRNA levels following treatment. qRT–PCR was used to determine the relative mRNA levels of *Aqp 3* after 12 h or 24 h in SB 220025. *Aqp 3* and *Aqp 9* mRNA was not significantly affected after 12 h; *n* = 3, mean ± SEM, *P*≤0.05. *Aqp 9* mRNA was not significantly affected after 24 h; however, *Aqp 3* mRNA was significantly decreased after 24 h. Each group is expressed relative to KSOMaa control. *n* = 3, mean ± SEM, *P*≤0.05. (B) AQP3 immunofluorescence after 24 h of p38 MAPK inhibition. AQP3 protein localization following treatment with KSOMaa, SB 202474, or SB 220025. Embryos treated with SB 220025 for 24 h displayed a clear and consistent reduction in AQP3 immunofluorescence compared to controls. Green  = AQP3; *n* = 10–15 embryos in each group; scale bar  = 10 microns.

We then analyzed the effects of blocking p38 MAPK activity on other genes involved in establishing and maintaining the trans-trophectoderm ion gradient during blastocyst formation by applying qPCR. We investigated the effects of SB 220025 treatment on *zonula occludens* (*Tjp1)*, *Occludin (Ocln),* and the *Na/K ATPase beta 1-subunit (Atp1b1*) mRNA levels [7], [8], [12], [18]. No significant difference in *Tjp1*, *Ocln,* or *Atp1b1* mRNA levels were observed among treatment groups (Figure 5). To complete our analysis of the effects of p38 MAPK inhibition on blastocyst formation gene families, we investigated the effects of SB 220025 treatment on *Aqp3* and *Aqp9* mRNA levels. After 12 h of SB 220025 treatment, there was no significant difference in *Aqp3* or *Aqp9* mRNA levels between treated and control groups (Figure 6A). After 24 h, *Aqp9* mRNA levels were unaffected; however *Aqp3* mRNA levels were significantly (p< 0.05) reduced in embryos treated with SB 220025 compared to controls (Figure 6A).

Protein staining as observed using confocal microscopy also revealed a similar pattern. Embryos treated for 12 h had little or no difference in AQP3 localization (data not shown). However, embryos treated for 24 h displayed an obvious and consistent reduction in AQP3 immunofluorescence intensity in p38 MAPK inhibited embryos (Figure 6B). AQP9 staining did not reveal any visible difference after either 12 h or 24 h time point (data not shown).

### Inhibition of p38 MAPK Signaling Increases Apoptosis in the Blastocyst

**Figure 7:**
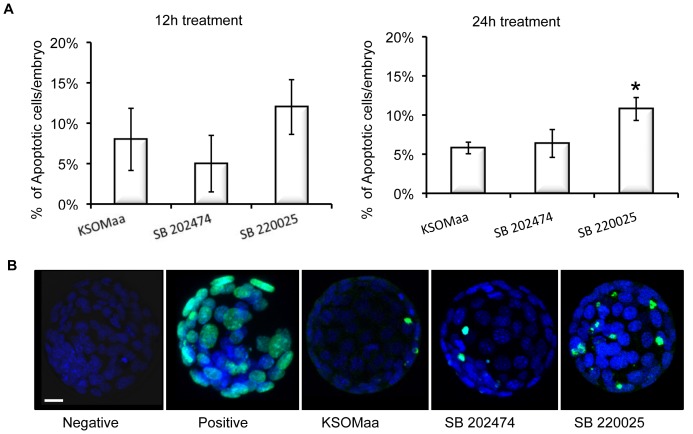
Prolonged inhibition of p38 MAPK increased apoptosis. (A) Apoptosis following KSOMaa, SB 202474 or SB 220025 treatment for 12 h and 24 h. TUNEL was used to assess the level of apoptosis in blastocysts after having been cultured in KSOMaa, SB 202474 or SB 220025 media for 12 h or 24 h. The number of individual cells that were TUNEL positive was counted in each blastocyst and is represented as the average number of cells that are TUNEL positive per blastocyst. Embryos cultured in KSOMaa, SB 202474 or SB 220025 for 12 h displayed the same average number of TUNEL positive cells. Embryos cultured in SB 220025 for 24 h displayed a significant increase in the average number of TUNEL positive cells compared with controls; mean ± SEM, *P*≤0.05. (B) Representative images of embryos from TUNEL assay. Negative: No fluorescence control; Positive: Embryos pretreated with DNAse to ensure TUNEL staining was successful. *n* = 4 replicates; 15–20 embryos in each group.

The results indicate that p38 MAPK regulates blastocyst expansion, zona hatching *in vitro*, TJ permeability, TJP1 localization and *Aqp3* mRNA levels. The timing of p38 MAPK inhibition is critical as 12 h treated embryos recovered from treatment as demonstrated by their resumption of cavitation following treatment and placement in drug free culture medium, while 24 h treated embryos did not recover from treatment. We thus investigated whether embryos treated for 24 h with SB 220025 were unable to recover due to increased induction of apoptosis. Blastocysts treated for 12 h with SB 220025 displayed no significant difference in apoptosis levels between groups (Figure 7A). However, embryos cultured for 24 h in SB 220025 displayed a significant (P<0.05) increase in apoptotic cells (11% ±1%, Figure 7A; representative images display in Figure 7B) over that observed for controls (KSOMaa (6% ±1%) and SB 202474 (6%±2%).

## Discussion

Preimplantation development is the most vulnerable period of mammalian development with the majority of human preimplantation embryos failing to reach the blastocyst stage or implant [35]. With the increasing reliance on fertility clinics and Assisted Reproductive Technologies (ARTs) to provide patients with an opportunity to have children [36]–[38], research focusing on understanding the mechanisms controlling preimplantation development and also on how the environment affects that developmental program is increasingly needed.

The Na/K-ATPase subunits, AJs, TJs and AQPs contribute directly to the mechanism that enables the TE to oversee cavitation and blastocyst formation (reviewed in [12], [25]. It is necessary to define the intracellular signaling mechanisms that regulate the functions of these gene products so that we can develop a comprehensive understanding of how cavitation and blastocyst formation are coordinated. The present study, with its focus on p38 MAPK, probed the role of this pathway and its contributions to cavitation and blastocyst formation.

We have demonstrated that p38 MAPK regulates cavitation and overall blastocyst formation. p38 MAPK inhibition in early blastocysts is accompanied by an increase in TJ permeability, shifts in TJP1 localization, down regulation of *Aqp3* mRNA levels, reduced AQP3 localization and increased apoptosis. These outcomes place p38 MAPK as a likely intracellular signaling mediator that coordinates the function of proteins involved in establishing the trans-trophectoderm ionic gradient during blastocyst formation. The consistency of outcomes observed regarding the effects to blastocyst cavity expansion for both p38 MAPK inhibitor treatments employed in this study, and the absence of observable effects to the ERK MAPK pathway during these treatments, support our conclusion that the effects we have observed are specifically attributable to a blockade of p38 MAPK activity and are not due to off-target effects from the inhibitors.

Gene deletion studies have demonstrated that p38 MAPK α is required for successful development with knockout mice suffering a developmental lethality by day 10.5 of pregnancy (reviewed in [39]). Each p38 MAPK inhibitor compound targets both p38 MAPK α and β forms and thus while preimplantation embryos may be able to complete this developmental interval in the absence of p38 MAPK α, we would suggest that our collective results demonstrate that targeting both p38 MAPK α and β using p38 MAPK inhibitors results in a cessation of preimplantation development and erosion of the adaptive mechanisms that early embryos use to adjust to the environment [22], [25]–[28]. This could indicate that p38 MAPK α and β may play overlapping or redundant roles during preimplantation embryonic development.

We were concerned that the outcomes on blastocyst expansion following p38 MAPK inhibition may be related to effects on TE differentiation or polarity. CDX2 activity is regulated by p38 MAPK phosphorylation in intestinal epithelial cell differentiation [40] and, p38 MAPK regulates *Cdh1* expression during gastrulation [41]–[43]. We therefore sought to determine if p38 MAPK regulated CDX2 and CDH1 localization in the blastocyst. We demonstrated no effect of p38 MAPK inhibition on CDX2 or CDH1 localization suggesting that TE differentiation and polarity were well established and likely unaffected by the p38 MAPK inhibition. It may be possible that p38 MAPK does affect TE differentiation and TE polarity but to determine this, experiments would have to be directed to earlier stages, prior to the onset of cavitation. The maintenance of TE differentiation and polarity ensure that the outcomes observed pertain specifically to p38 MAPK regulation of cavitation and are not due to disruption of TE differentiation or polarity.

We also concluded that F-actin was present and properly localized in p38 MAPK inhibited embryos. We did not observe any changes in staining intensity of F-actin in p38 MAPK inhibited blastocyst unlike those reported by Natale *et* *al*. 2004 and Paliga *et* *al*., 2005. Natale *et* *al*., 2004, and Paliga *et* *al*., 2005, demonstrated that p38 MAPK signaling was required at the 8–16 cell stage for normal blastocyst development through its regulation of heat-shock protein 25/27 which stabilizes F-actin. F-actin stability was restored after p38 MAPK inhibition was removed and embryos were able to progress to the blastocyst stage [22], [26]. The differences we have observed between our two studies could indicate a stage specific role that p38 MAPK plays at the 8–16 cell stage versus the blastocyst stage. This result may have revealed a developmental maturation that p38 MAPK undergoes as the embryo advances towards the blastocyst stage and prepares for implantation.

We observed an effect of p38 MAPK inhibition treatment time on blastocyst cavitation and zona hatching. Both a 12 h and 24 h treatment time had a significant negative effect on blastocyst cavitation and zona hatching *in vitro*. Embryos treated for 12 h displayed an ability to resume expansion after treatment while those treated for 24 h did not. Both treatment times resulted in a blockade of zona hatching *in vitro* even after placement in fresh culture drops and allowed to recover. Our interpretation of these outcomes is that the 12 h treatment slowed down the developmental program sufficiently to prevent the embryos from fully catching up to controls, which had hatched, during the recovery period. This outcome mirrors our observations of the effects of p38 MAPK inhibition treatment on cleavage stage embryos, where they proceeded to the blastocyst stage in normal frequencies once p38 MAPK inhibition treatment is removed, albeit in a delayed fashion [22], [26]. Alternatively, we suggest that the 24 h treatment was long enough to deprive the embryo its adaptive mechanism to respond to environmental stress resulting in an inability of the embryo to recover (Reviewed in [21], [22], [25]–[27], [39]. The down regulation of *Aqp3* mRNA levels and reduced AQP3 detection observed only at the 24 h treatment time is certainly consistent with this interpretation of our results. A decline in *Aqp3* mRNA levels and reduced AQP3 detection are suggestive that facilitated fluid movement across the TE may be impeded by 24 h p38 MAPK inhibition treatment. If so, this would certainly contribute to the inability of 24 h p38 MAPK inhibition treated embryos to resume expansion and is consistent with our earlier report that p38 MAPK regulates *Aqp* mRNAs levels in cleavage stage embryos [25]. Apoptosis was unaffected by the 12 h p38 MAPK inhibition treatment period, but the 24 h treatment period was accompanied by a significant increase in apoptosis. Studies have demonstrated that it is the JNK/SAPK pathway that regulates apoptosis in the preimplantation embryo, especially following environmental stress [25], [44]–[46].We propose that failure to resume cavitation, increased apoptosis, and the loss of AQP3 are reflective of the effects of environmental stress on the embryo due to the prolonged inhibition of the p38 MAPK pathway [25], [27].

Finally, the increased FITC-dextran permeability we observed following p38 MAPK inhibition treatment is consistent with our earlier studies [8], [12]. Loss of p38 MAPK signaling during EMT resulted in disorganized and loss of expression of TJP1 in human mesothelial cells [47]. Collectively, these studies provide an understanding of the mechanisms that control TJ function during cavitation and reinforce our understanding of the role the TJ permeability seal plays in regulating cavitation and blastocyst formation [8], [12], [18]. In conclusion, our results support an important role for p38 MAPK in mediating cavitation and blastocyst expansion and suggest that it regulates blastocyst formation gene family expression, function and thus coordination of the overall events that control blastocyst formation.

## Materials and Methods

### Ovulation Induction and Mouse Embryo Collection

Female MF-1 mice (Charles River, Canada; 3–5 weeks old) were injected with 5 IU Pregnant Mare's Serum Gonadotropin (PMSG)(Intervet Canada Ltd, Whitby, Ontario, Canada), followed by 5 IU human Chorionic Gonadotropin (hCG) (Intervet) 48 h later and mated with CD-1 males. Successful mating was determined the following morning (Day 1) by the detection of a vaginal plug. Time post-hCG was used to measure the developmental stage of the embryos. Preimplantation mouse embryos were flushed from the reproductive tract using M2 flushing media (Sigma, St Louis, MO, USA) at 89 h (early blastocyst) post-hCG. All animal care and embryo collection methods were conducted using approved protocols from the Canadian Council of Animal Care and the University of Western Ontario Animal Care and Veterinary Services. The approved University of Western Ontario animal care protocol number for these studies is 2010-021Watson.

### Embryo Culture and Treatment

#### Concentration Response Experiments

Embryos were washed four to five times in 50 µl drops of either potassium simplex optimized medium with amino acids (KSOMaa, Chemicon, Temecula, CA, USA) +0.2% dimethylsulfoxide (DMSO), KSOMaa +20 µM SB 202474 (inactive analogue control), KSOMaa + 2 µM SB 220025, KSOMaa + 5 µM SB 220025, KSOMaa +10 µM SB 220025 or KSOMaa + 20 µM SB 220025 and transferred to 20 µl drops of the respective treatment under light paraffin oil. They were maintained in culture under 5% CO_2_ in air atmosphere at 37°C for 24 h. Embryos were imaged at 4 h, 8 h, 12 h and 24 h post collection. After 12 h or 24 h of treatment, embryos from each group were washed in fresh KSOMaa (minimum of 3 times) and placed in fresh KSOMaa 20 µl culture drops for recovery experiments. Embryos were imaged at time points equivalent to 24 h, 28 h and 36 h post embryo collection. These time points are equivalent to 12 h, 16 h and 24 h of recovery for 12 h treated embryos and 4 h and 12 h recovery for 24 h treated embryos respectively (Figure S4). Embryo diameter was used to assess blastocyst expansion. Diameter of each blastocyst was measured in two different directions (using Image Pro analysis 6.2 software; Figure S5) and then averaged. To confirm expansion results from embryos treated with SB 220025, embryos were also treated with 5 µM SB 203580, a second potent p38 inhibitor, for 24 h and measured.

#### Assessment of Zona Hatching *In Vitro*

Embryos were washed four to five times in 50 µl drops of treatment medium and transferred to 20 µl drops of the respective treatment medium under light paraffin oil. They were maintained in culture under 5% CO_2_ in air atmosphere at 37°C for 12 h or 24 h. Embryo hatching was assessed at 24 h and 48 h post collection. An embryo was considered to be “hatching” if any part of the embryo protruded from the zona pellucid, or “hatched” if the embryo was completely free of the zona pellucida. Four replicates were performed.

### Quantitative RT-PCR

Quantitative RT-PCR (qRT-PCR) was conducted to determine the effects of p38 MAPK inhibition on the mRNA levels of TE associated proteins. Total RNA was extracted from pools of 20 embryos using the PicoPure kit (Arcturus, Molecular Device, Sunnyvale, CA). Reverse Transcription (RT) reaction was carried out using Sensiscript reverse transcriptase (Qiagen, Mississauga, ON). The samples were incubated in 10x buffer, RNAse inhibitor, dNTPs and random primers at 37°C for 1 h. Q RT-PCR reactions were performed using the BioRad Chromo4 detection system (BioRad, Mississauga, ON). PCR was carried out in 20 µl reactions containing 10 µl SYBR Green (Invitrogen, Burlington, ON), 0.2 µl of primer sets provided by Biosearch Technologies (Novado, CA), 1 µl of appropriate dilution of cDNA (1 embryo/µl), and 8.8 µl of water. To confirm the specificity of each quantitative RT-PCR product, amplicons were extracted from gels and purified using a QIAquick gel extraction kit (Qiagen, Mississauga, ON) and submitted for nucleotide sequencing (DNA Sequencing Facility, Robarts Research Institute, London, ON, Canada). The nucleotide sequences were then compared with sequences available in GenBankTM nucleotide sequence data base, and in all cases the specificity of each PCR product was confirmed. *Aqp3* and *Aqp9* were obtained from Applied Biosystems (*Aqp3*Assay ID: mm01208559_m1; *Aqp 9* Assay ID: mm00508094_m1). All other sequences can be found in Figure S6. Three to six replicates were performed.

### Antisera

Antibodies used in whole mount immunofluorescence procedures were obtained from commercial sources: CDH1 and TJP1 (Upstate Cell Signaling Solutions, Charlottesville, VA), CDX2 (Aviva Systems Biology, San Diego, CA), AQP3 and 9 (Alpha Diagnostic Int. (San Antonio, TX, USA)), ERK and p-ERK (Cell Signalling Technologies (Danvers, MA, USA).

### Indirect Immunofluorescence

Mouse embryos were collected from the reproductive tracts of super ovulated female MF-1 mice as described above. Embryo pools were washed in 1x PBS (GIBCO) and fixed for indirect immunofluorescence in 2% paraformaldehyde in PBS at room temperature for 30 minutes. Fixed embryos were washed once in 1× PBS and either used immediately or stored at 4°C in embryo storage buffer (1× PBS +0.1% sodium azide) for up to 1 week before processing for whole-mount indirect immunofluorescence as previously described [16], [18], [27], [29], [30]. Fixed embryos were permeabilized in blocking buffer (0.01% Triton X-100 +5% normal donkey serum in 1× PBS) at room temperature for 1 h followed by two washes in fresh PBS. Embryos were incubated with primary antibody in a 1∶100 dilution in antibody dilution/wash buffer (ADB; 0.005% Triton X-100 +0.5% normal donkey serum in 1× PBS) at 4°C overnight. Negative controls included embryos incubated in ADB alone without the addition of the primary antibody in order to determine the levels of non-specific fluorescence. Embryos were then washed three times for 30 minutes in ADB at 37°C and incubated with fluorescein isothiocyanate (FITC)-conjugated secondary antibody (1∶200 in ADB) at 4°C overnight, followed by three washes for 30 minutes in ADB at 37°C. 4′,6-diamidino-2-phenylindole (DAPI; Sigma-Aldrich; diluted 1∶2000 from 1 mg/ml stock solution), a nuclear stain, along with Rhodamine phalloidin (diluted 1∶20 into ADB), a filamentous actin (F-actin) stain, were added to the first wash step. Fully processed embryos were mounted onto glass slides in 20 µl of Vectashield mounting medium (Vector, Burlingame, CA) under elevated 22×22 mm glass cover slips (No. 1 thickness), and slide preparations were sealed with nail polish. Slides were stored for up to 2 days at 4°C in a light tight box prior to immunofluorescence imaging. Embryos were examined by confocal microscopy using an Olympus Fluoview 1000 laser scanning Confocal Microscope (Olympus, Canada). In total, 10–15 embryos for each treatment were examined for each of the primary antisera.

### Assessment of TJ Function by FITC-Dextran Uptake Assay

To investigate the effects of p38 MAPK inhibition on TJ permeability, embryos were cultured for 12 h or 24 h in KSOMaa, SB 202474, SB 220025 or for 30 minutes in 2 mM EGTA (ethylene glycol Bis- (β-aminoethyl ether) N,N,N,N -tetraacetic acid). After treatment, blastocysts were cultured in drops containing 40 kDa FITC-Dextran in KSOMaa for 30 minutes. Following this incubation, blastocysts were immediately washed in three 50 µL wash drops of KSOMaa and placed in a fourth clean KSOMaa drop for immediate assessment of FITC-dextran accumulation employing an epi-fluorescent microscope (Olympus). Three replicates were performed for each time point.

### TUNEL Apoptosis Assay

Cleavage of genomic DNA during apoptosis yields single strand breaks in high molecular weight DNA, which can be identified by labeling the free 3′-OH terminal with modified nucleotides in an enzymatic reaction [31], [32]. After 24 h of treatment, embryo pools were fixed for 1 h in 2% paraformaldehyde in PBS and washed in PBS. Fixed embryos were then permeabilized and blocked for 3 minutes in permeabilization solution (1% sodium citrate +0.01% Triton X-100) at 4°C. Embryos were then washed in PBS, and a positive control group was removed from the treatment groups and treated with 100 µl DNAse solution (10 µl DNAse1 (Invitrogen) in 990 µl 50 mM Tris-HCl) for 13 minutes at room temperature. This was done to induce strand breaks, allowing for the assessment of maximal DNA fragmentation during analysis. A group of embryos was removed from the treatment groups to serve as the negative control group. The positive control and the treatment groups were placed in 50 µl TUNEL reaction mixture (terminal deoxynucleotidyl transferase from calf thymus, recombinant in *Escherichia coli*, in storage buffer + nucleotide mixture in reaction buffer). The TUNEL reaction mixture labeled DNA strand breaks through terminal deoxynucleotidyl transferase, which catalyzes polymerization of labeled nucleotides to the free 3′-OH DNA ends in a template-independent manner, and by incorporating fluorescein labels in nucleotide polymers [31], [33]. Negative control embryos were put into 50 µl TUNEL label solution (nucleotide mixture in reaction buffer) only. Embryos were incubated for 60 minutes in a humidified chamber at 37°C. The groups were then washed in 0.5 µl DAPI (Sigma-Aldrich; diluted 1∶2000 from 1 mg/ml stock solution) +200 µl PBS for 30 minutes, to stain the DNA. The groups were washed twice more in 200 µl PBS, for 15 minutes at 37°C. Fully processed embryos were mounted onto glass slides in a drop of Vectashield mounting medium. Three replicates were performed for each time point.

### Apoptotic Fluorescence Image Analysis

Embryos were examined by confocal microscopy using an Olympus Fluoview 1000 laser scanning Confocal Microscope (Olympus, Canada). Z-stack images were taken of the embryos and used to count total cell number and FITC positive cell number. Apoptotic fluorescence was assessed by first counting the number of DAPI-stained (blue) nuclei, representing the total number of cells in the early embryo followed by counting the FITC-stained (green) apoptotic nuclei. To obtain the percent of apoptotic cells, the number of FITC-stained cells was divided by the number of DAPI-stained cells. All microscope and image capture settings remained constant during the digital capture of micrographs between embryos and between treatment groups. 10–15 embryos were assessed for each treatment group.

### Statistical Analysis

The results from the concentration response and recovery experiments were first subjected to Normality tests and once passed, analyzed by a repeated measure ANOVA test followed by a Student-Newman-Keuls post hoc test. All other experiments were also first subjected to Normality tests and once passed, analyzed by a one-way ANOVA test followed by a Student-Newman-Keuls post hoc test. All error bars represent standard error mean (SEM). Significant p value ≤0.05. Data was analyzed using the SigmaStat® 3.5 (Jandel Scientific Software, San Rafael, CA, USA).

## Supporting Information

Figure S1 Representative images of embryos from each treatment group at each time point during the recovery experiment. Scale bar  = 20 microns.
(TIF)

Figure S2 Embryos were cultured for 24 h in control media (KSOMaa and SB 202474) or in KSOMaa + SB 203580 and imaged at 0 h, 12 h, and 24 h of treatment. The diameter of each embryo was measured as described in Figure S5. After 24 h embryos cultured in SB 203580 were significantly less expanded than controls. Three replicates were performed and 15–20 embryos were measured in each group. ± SEM; p≤0.05.
(PDF)

Figure S3 Total-ERK and phospho–ERK protein following treatment with KSOMaa, SB 202474, or SB 220025 using immunofluorescense. Representative images of total-ERK after 24 h of treatment KSOMaa (A), SB 202474 (B) or SB 220025 (C) and phospho-ERK after 24 h of treatment KSOMaa (D), SB 202474 (E) or SB 220025 (F). There was no visible difference between total-ERK or phospho-ERK between the treatment groups. Green  =  total-ERK (A–C); Green  =  phosphor-ERK (D–F); No primary (G) *n* = 10–15 embryos in each group; scale bar  = 10 microns.
(TIF)

Figure S4 Timeline for embryo collection, treatment time, and recovery time for all experiments. Embryos were collected at 89 h post hCG and cultured for 12 h or 24 h. Embryos were imaged and measured at 0 h, 4 h, 8 h, 12 h, and 24 h post hCG, and again following recovery, at 24 h, 28 h and 36 h post hCG. Embryos were collected at 12 h or 24 h and either analyzed for RT-PCR, FITC assay, TUNEL assay or immunofluorescense or placed into fresh culture drops and allowed to recover. Hatching was assessed at 24 h post hCG.
(TIF)

Figure S5 Embryo diameters were measured for dose response and recovery experiments. Embryos were measured in two different directions and then averaged.
(TIF)

Figure S6 Quantitative RT-PCR primer sets.
(TIF)
