# Supplementary figures and images for: p38 MAPK Regulates Cavitation and Tight Junction Function in the Mouse Blastocyst
Source: PLoS One. 2013 Apr 4;8(4):e59528. doi: 10.1371/journal.pone.0059528 (PMC3617173; doi:10.1371/journal.pone.0059528)

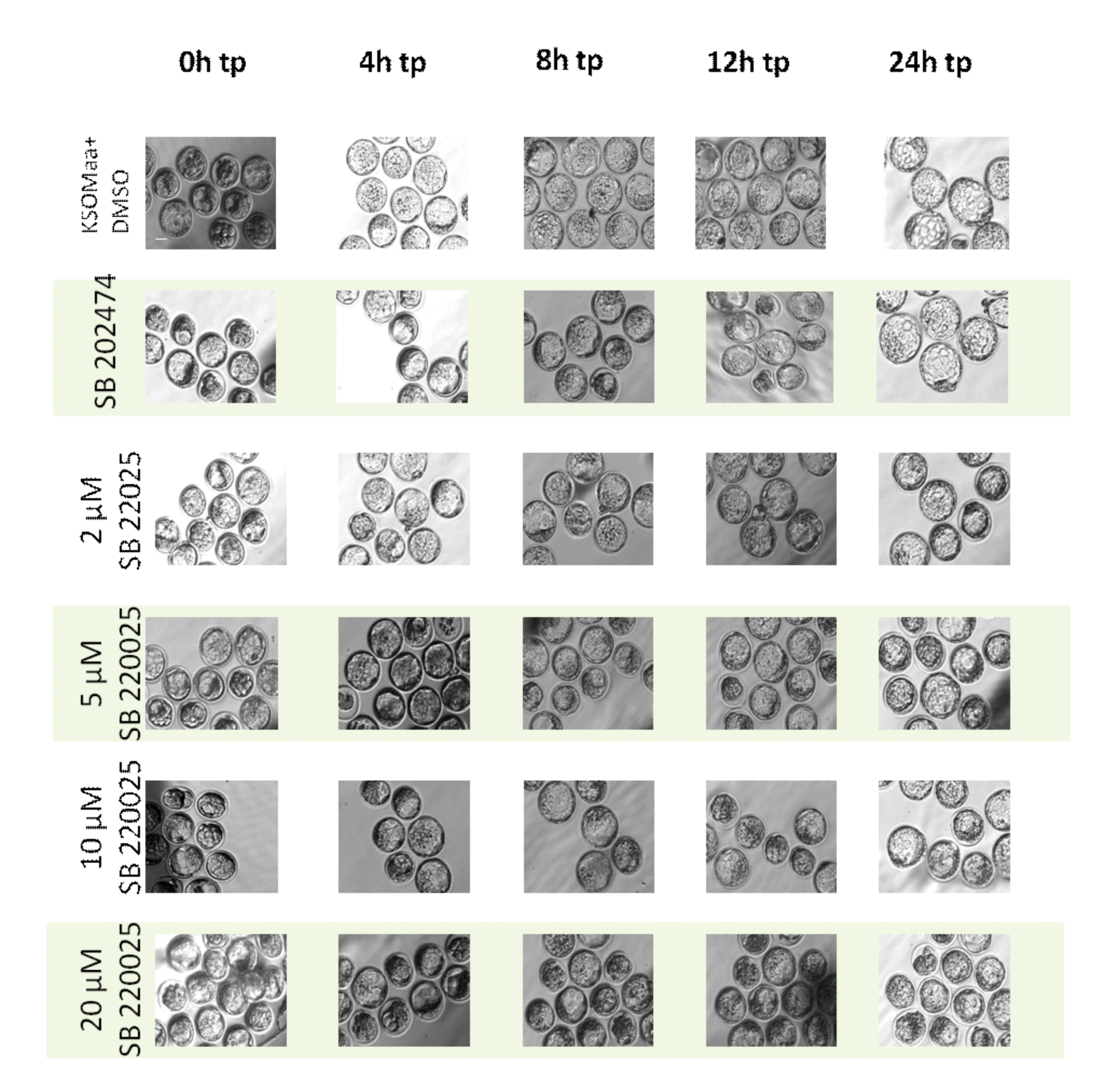

Supplement: Figure S1 — Representative images of embryos from each treatment group at each time point during the recovery experiment. Scale bar = 20 microns. (TIF) [file pone.0059528.s001.tif]

Supporting Figure 2

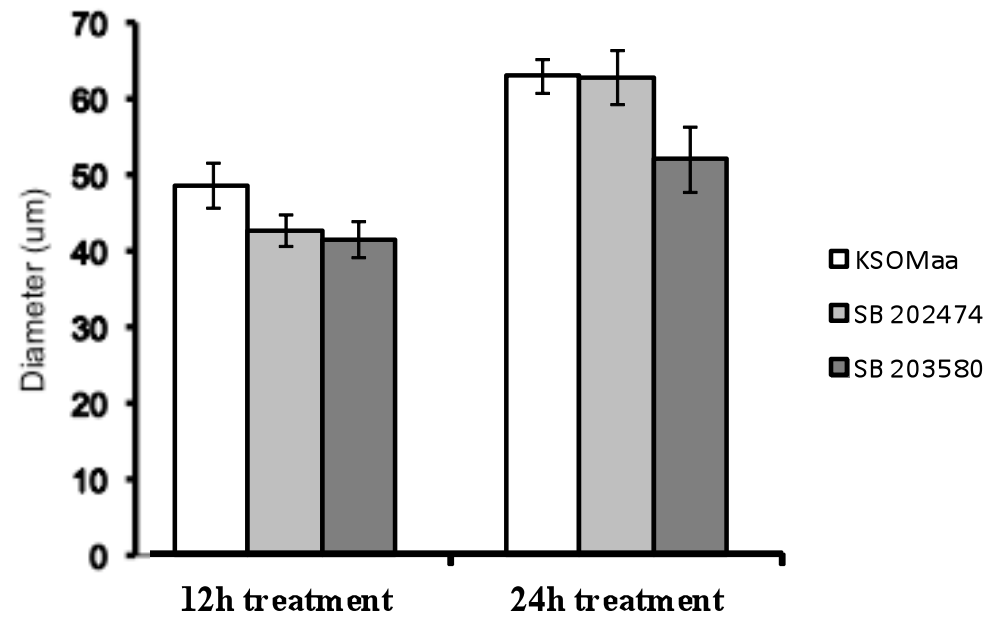

Supplement: Figure S2 — Embryos were cultured for 24 h in control media (KSOMaa and SB 202474) or in KSOMaa + SB 203580 and imaged at 0 h, 12 h, and 24 h of treatment. The diameter of each embryo was measured as described in Figure S5. After 24 h embryos cultured in SB 203580 were significantly less expanded than controls. Three replicates were performed and 15–20 embryos were measured in each group. ± SEM; p≤0.05. (PDF) [file pone.0059528.s002.pdf]

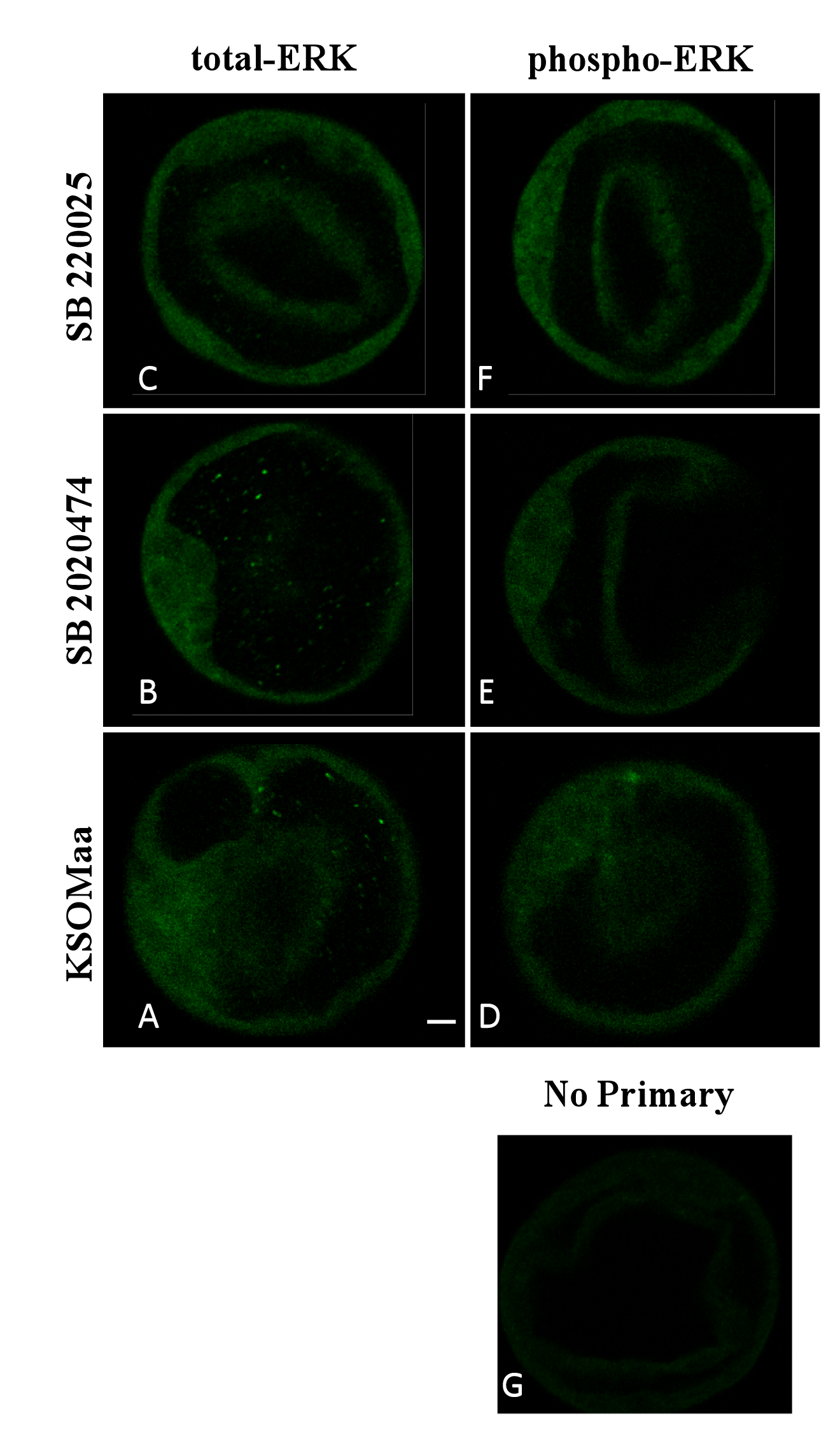

Supplement: Figure S3 — Total-ERK and phospho–ERK protein following treatment with KSOMaa, SB 202474, or SB 220025 using immunofluorescense. Representative images of total-ERK after 24 h of treatment KSOMaa (A), SB 202474 (B) or SB 220025 (C) and phospho-ERK after 24 h of treatment KSOMaa (D), SB 202474 (E) or SB 220025 (F). There was no visible difference between total-ERK or phospho-ERK between the treatment groups. Green = total-ERK (A–C); Green = phosphor-ERK (D–F); No primary (G) n = 10–15 embryos in each group; scale bar = 10 microns. (TIF) [file pone.0059528.s003.tif]

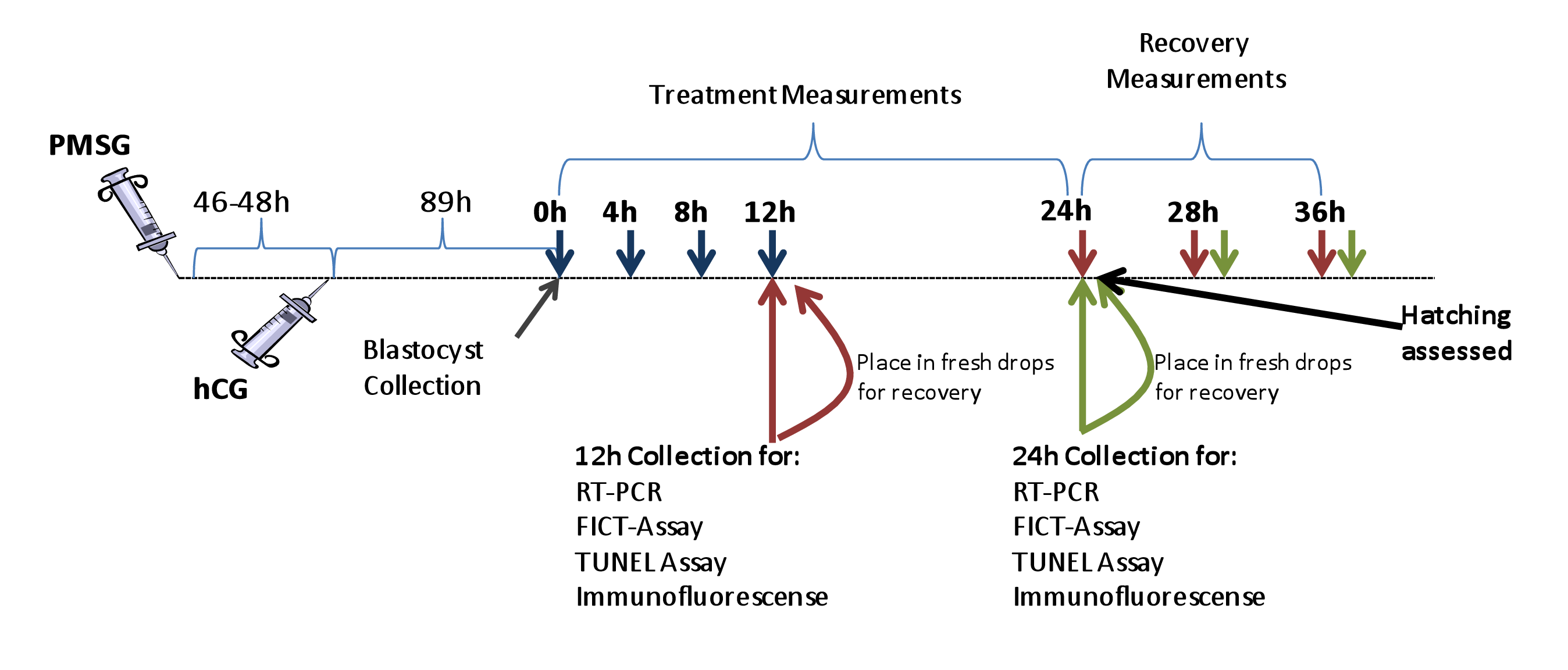

Supplement: Figure S4 — Timeline for embryo collection, treatment time, and recovery time for all experiments. Embryos were collected at 89 h post hCG and cultured for 12 h or 24 h. Embryos were imaged and measured at 0 h, 4 h, 8 h, 12 h, and 24 h post hCG, and again following recovery, at 24 h, 28 h and 36 h post hCG. Embryos were collected at 12 h or 24 h and either analyzed for RT-PCR, FITC assay, TUNEL assay or immunofluorescense or placed into fresh culture drops and allowed to recover. Hatching was assessed at 24 h post hCG. (TIF) [file pone.0059528.s004.tif]

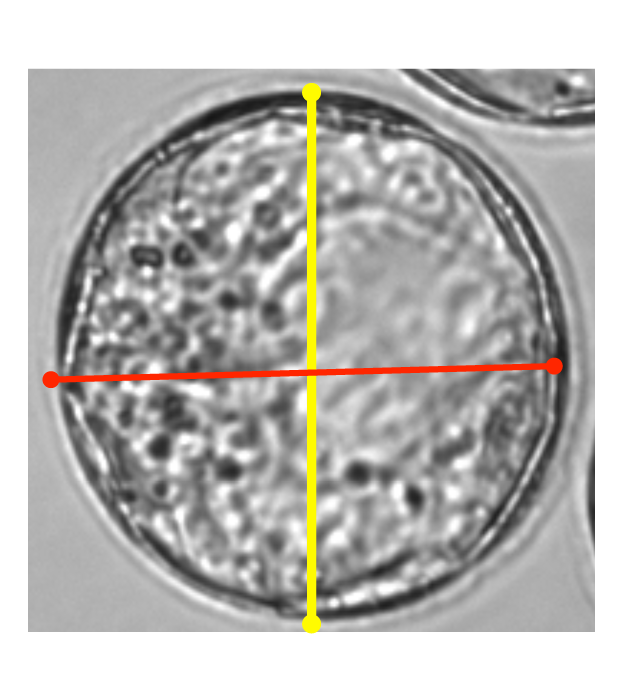

Supplement: Figure S5 — Embryo diameters were measured for dose response and recovery experiments. Embryos were measured in two different directions and then averaged. (TIF) [file pone.0059528.s005.tif]

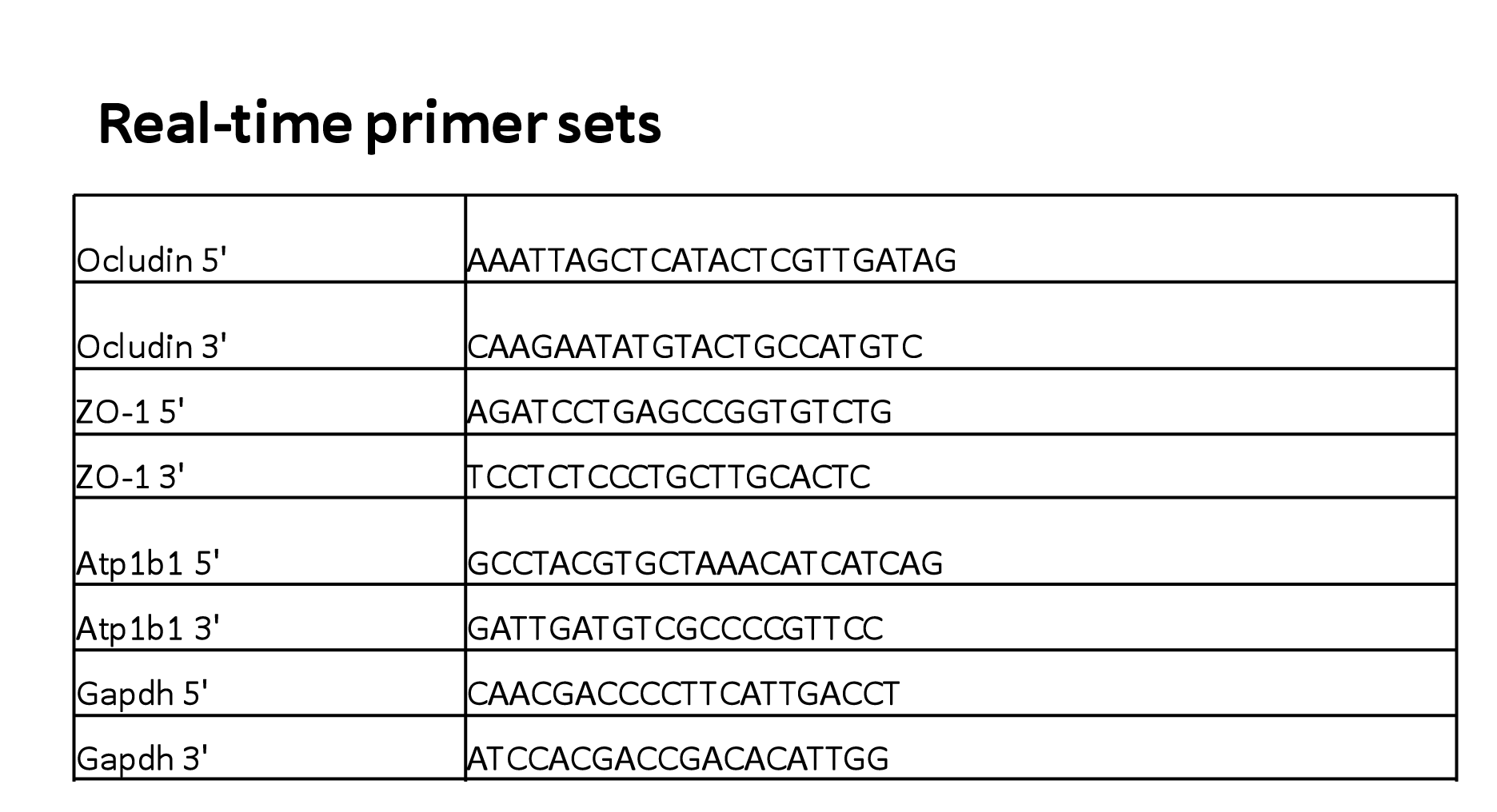

Supplement: Figure S6 — Quantitative RT-PCR primer sets. (TIF) [file pone.0059528.s006.tif]
